# Correcting *SUVR* bias by accounting for radiotracer clearance in tissue: A validation study with [^18^F]FE-PE2I PET in cross-sectional, test-retest and longitudinal cohorts

**DOI:** 10.1177/0271678X251322407

**Published:** 2025-02-21

**Authors:** Minyoung Oh, Praveen Honhar, Richard E Carson, Ansel T Hillmer, Andrea Varrone

**Affiliations:** 1 Department of Clinical Neuroscience, Centre for Psychiatry Research, Karolinska Institutet and Stockholm Health Care Services, Stockholm, Sweden; 2 Departments of Nuclear Medicine, 65526 Asan Medical Center, University of Ulsan College of Medicine, Seoul, Republic of Korea; 3 Department of Radiology and Biomedical Imaging, Yale PET Center, Yale School of Medicine, New Haven, CT, USA; 4 Department of Biomedical Engineering, Yale University, New Haven, CT, USA; 5 Department of Psychiatry, Yale School of Medicine, New Haven, CT, USA

**Keywords:** Dopamine transporter, DVR, PET quantification, SUVR, tissue clearance correction

## Abstract

Quantification of dopamine transporter (DAT) with [^18^F]FE-PE2I PET is an important progression marker for Parkinson’s disease (PD). This study aimed to validate a novel correction (*SUVR*c) for a less-biased estimate of *SUVR* by accounting for [^18^F]FE-PE2I clearance-rate, in independent cross-sectional (38 PD, 38 controls), test-retest (9 PD) and longitudinal cohorts (21 PD). *SUVR*c was calculated as $\frac{\mathrm{SUVR}}{1\;-\;\frac{\beta_{\mathrm{ref}}}{k_{2,\mathrm{ref}}}\;+\beta_{\mathrm{tar}}\frac{\mathrm{SUVR}}{k_{2,\mathrm{ref}}R_{1}}}$ . *β*_tar_ and *β*_ref_ are the clearance rates from the target and reference tissues. Bias relative to *DVR*, discriminative power, test-retest variability (TRV) and annual longitudinal change (ALC) were used to compare *SUVR*_50–80 min_, *SUVR*c_50–80 min_, *SUVR*_15–45 min_ and *DVR. SUVR*_50–80 min_ showed high bias across all regions (HC: mean: 48.31 ± 20.49% [range: 28.32–53.80%]; PD: 29.91 ± 13.95% [20.45–39.80%]) that was corrected by *SUVR*c_50–80 min_ (HC: −0.80 ± 12.72% [−9.69–11.64%]; PD: −0.13 ± 7.41% [−5.04–2.97%]), *p < *0.001 for both groups compared to mean bias of *SUVR*_50–80 min_, similar to *SUVR*_15–45 min_. For the striatum, Cohen’s *d* was similar for all measures. TRV were 3.2 ± 2.5% (*DVR*), 6.4 ± 5.7% (*SUVR*_50–80 min_), 6.8 ± 5.9% (*SUVR*c_50–80 min_) and 3.9 ± 3.2% (*SUVR*_15–45 min_). Higher TRV of *SUVR*c_50–80 min_ was due to TRV of 9.2 ± 5.1% [1.1–19.4] for β_tar_. ALC was 4.5 ± 4.2% (*DVR*), 5.2 ± 6.5% (*SUVR*_50–80 min_), 4.4 ± 4.1% (*SUVR*c_50–80 min_) and 4.2 ± 4.1% (*SUVR*_15–45 min_). *SUVR*c_50–80 min_ reduced bias compared to *SUVR*_50–80 min_, as previously reported. *SUVR*c_50–80 min_ was sensitive to small changes of β_tar_, with higher TRV compared to *DVR*, but with similar ALC, suggesting that it can reliably assess longitudinal DAT changes.

## Introduction

Parkinson’s disease (PD) is a neurodegenerative disease characterized by the selective degeneration of dopaminergic neurons in the pars compacta of the substantia nigra (SN) and striatal terminals, with reduction of the dopamine transporters (DAT). DAT imaging plays a crucial role in the assessment of nigrostriatal degeneration in patients with Parkinsonism as it provides *in vivo* assessment of presynaptic dopaminergic system integrity. [^18^F]FE-PE2I([^18^F]-(E)-N-(3-iodoprop-2-enyl)-2β-carbofluoroethoxy-3β- (4′-methyl-phenyl) nortropane) is a positron emission tomography (PET) ligand with high affinity and selectivity for DAT.^1^ The better spatial resolution of PET vs. SPECT^2,3^ and faster kinetic properties compared to [^11^C]PE2I,^4^ make [^18^F]-FE-PE2I PET a suitable tool for DAT imaging and quantification, which could improve on DAT SPECT in clinical practice.

The use of a simplified reference tissue model (SRTM) derived parameters, including binding potential relative to non-displaceable radioligand in tissue (*BP*_ND_) or distribution volume ratio (*DVR*), for DAT quantification with [^18^F]FE-PE2I has already been validated in several studies.^5–8^ Compartment modeling, however, requires long scan-time to acquire dynamic data, which is less feasible in a clinical practice, particularly in patients with neurodegenerative disorders. An alternative approach to reduce scan-time for [^18^F]FE-PE2I PET has been investigated.^7,8^ It makes use of standardized uptake value ratio (*SUVR*), defined as the ratio of activity concentration in a target tissue to reference tissue during a specified time-window. *SUVR* is generally well-correlated with *BP*_ND_ or *DVR*, but is usually biased, overestimating *DVR* at late time window^5,7,8^ due to radioligand clearance in tissue.^9^

Recently, Honhar et al. proposed a method that improves *SUVR* quantification by correcting for radioligand clearance in tissue (corrected *SUVR* or *SUVR*c). Their method demonstrated reduction of mean *SUVR* bias across regions and subjects for [^18^F]FE-PE2I in 40–60 minutes cross-sectionally.^10^ The detailed derivation of the correction for *SUVR* has been reported.^10^ In brief, *SUVR*c is calculated as follows (equation (1)).

(1) $$\mathrm{SUVRc}=\frac{\mathrm{SUVR}}{1-\frac{\beta_{\mathrm{ref}}}{k_{2,\mathrm{ref}}}+\beta_{\mathrm{tar}}\frac{\mathrm{SUVR}}{k_{2,\mathrm{ref}}R_{1}}}$$

The denominator on the right-hand side of equation (1) represents the correction needed in *SUVR* due to non-equilibrium tracer kinetics from the reference and target regions. Note that if the tracer was at true equilibrium or steady-state kinetics, β_*ref*_ = β_*tar*_ = 0, and we would have the trivial result that *SUVR*c = *SUVR* (no correction needed). Two terms in the denominator correct for the effects of tracer clearance on *SUVR*. For example, a faster clearance of the radiotracer in the target tissue compared to reference tissue leads to a more positive bias in *SUVR* (numerator), which is then corrected by a higher value of the denominator due to the third term, and vice-versa. R_1_ and k_2,ref_ can be approximated a *priori* based on previously published literature estimates.^10^ In this work we utilized the average SRTM k_2_′ as the k_2,*ref*_ and R_1_ = 1, consistent with a previous study.^10^ However, simulations of [^18^F]FE-PE2I dynamics in the putamen of HC and PD participants with varying blood-flow (R_1_) were studied (Supplementary Figure 1) to characterize the errors in corrected *SUVR* due to the *a priori* choice of R_1_ = 1. β_tar_ and β_ref_ are the clearance rates of the ligand from the target and reference regions during *SUVR* time-window, respectively. β_ref_ was estimated from a one-exponential fit to cerebellar time-activity curve during *SUVR* time-window.^10^ Direct estimation of β_tar_ from just *SUVR* time-window data is extremely sensitive to noise, especially in smaller regions or voxels. Therefore, β_tar_ is estimated from a previously-described linear regression^10^ that expresses β_tar_ as a function of a composite region’s (a relatively larger region with high uptake, here defined as combination of the caudate and putamen) clearance rate during *SUVR* time-window, and the difference between ${\mathrm{SUVR}}_{\mathrm{composite}}$ and ${\mathrm{SUVR}}_{\mathrm{tar}}$ (equation (2)).

[2] $$\beta_{\mathrm{tar}}=\beta_{\mathrm{composite}}+a\left(\frac{1}{{\mathrm{SUVR}}_{\mathrm{tar}}}-\frac{1}{{\mathrm{SUVR}}_{\mathrm{composite}}}\right)$$

While equation (2) has been conceived empirically, it underscores the inverse relationship between the target availability (in *SUVR* units) within a tissue and the clearance of the radiotracer within it, i.e., the radiotracer should have a smaller clearance rate in tissue with higher target availability. Using this idea, the clearance rate of the tracer from any brain tissue can be expressed as a linear function of a known clearance rate from a composite tissue, and the difference between the target availability (in inverse of *SUVR* units) amongst the two tissue types. This does assume the existence of a composite region for which the tracer clearance rate is known or can be reliably estimated from data collected within *SUVR* time-window, which in practice is true for relatively large regions with high tracer uptake. Note that this regression estimator contains a parameter called the radiotracer constant, a, which depends on the radiotracer and time-window for *SUVR.* This constant must be estimated prior to application. In this work, the estimate of a was optimized from a previously reported [^18^F]FE-PE2I PD cohort at Yale University’s PET center^10^; a = 0.0024 min^−1^ was used in equation (2) for *SUVR*c results reported for the *SUVR* time-window 50–80 min. The same leave-one-out procedure described in that work^10^ was utilized here. Briefly, 20 PD participants for whom dynamic PET data were acquired in that study, were divided into training and test subjects using a leave-one-out approach. For the 19 subjects in the training set, *SUVR*_*tar*_ (for a set of four regions: caudate, putamen, substantia nigra, ventral striatum), *SUVR*_*composite*_ and β_*composite*_ were computed (composite = striatum). Further, for the subjects within the training set β_*tar*_ was also estimated during *SUVR* time-window by utilizing low noise SRTM fits to the full dynamic data. Using ordinary least squares, parameter ***a*** was estimated, which was then used for the remaining subject to estimate β_*tar*_, that was in turn used to perform *SUVR* correction. The final optimized value of ***a*** was computed as the average value of all estimates during the leave-one-out approach.

In this study, we validated *SUVR*c in independent cross-sectional, longitudinal, and test-retest cohorts of [^18^F]FE-PE2I PET in HC and PD. The outcome measure of interest was the corrected *SUVR* using data at a late acquisition time-window (50–80 min). We compared diagnostic performance of *SUVRc_50–80 min_* with *DVR* and early *SUVR* (15–45 min), which has been shown to be one of the best time-windows, offering strong correlation with DVR, relatively low bias, and good reproducibility, as well as reliable longitudinal assessment in previous reports.^8^

## Material and methods

### Patients

The participants included in this study were part of three studies approved by the Ethics Committee of the Stockholm Region, by the Swedish Ethical Review Authority, by the Radiation Safety Committee of the Karolinska University Hospital, Stockholm, Sweden, and by the Swedish Medicinal Product Agency. The studies were registered as Clinical Trials in the EudraCT database: EudraCT 2011-002005-30 (EPN Dnr: 2011/703-31/2), EudraCT 2017-001585-19 (EPN Dnr: 2017/878-31/4), and EudraCT 2017-003327-29 (EPN Dnr: 2017/1605-31). The studies were conducted according to the ethical standards of the Ethics Committee of the Stockholm Region and the Swedish Ethical Review Authority and were in line with the Declaration of Helsinki. Patients provided written informed consent for study participation after detailed explanation from the investigator.

Demographic and clinical data are summarized in Table 1. In short, the total cohort consists of thirty-eight PD patients (median age, 67 years; range: 45–79 years) and thirty-eight age- and sex-matched healthy controls (median age, 66 years; range: 43–74 years). The cross-sectional, test-retest (n = 9, median age, 68 years; range: 54–74 years) and longitudinal (n = 21, median age, 67 years; range: 47–74 years) DAT PET data for the three cohorts have already been reported.^11–13^ All participants underwent the same screening procedure, i.e. exclusion of clinically relevant comorbidities, psychiatric conditions, illicit drug abuse or alcoholism, as assessed by structured interview, physical examination, blood tests, electrocardiogram, and brain MRI. Mini-Mental State Examination was performed to exclude cognitive decline. PD patients fulfilled the clinical diagnosis of PD according to the UK Parkinson’s Disease Brain Bank criteria.^14^ The re-analysis of the data with the new method was approved by the Swedish Ethical Review Authority.

**Table 1.** Demographics and clinical characteristics.

		Sex		Age (yr)		Symptom duration (yr)		H&Y	
	Numbers	F	M	median	range	median	range	median	range
HC	38	11	27	66	43–74				
PD cross-sectional	38	10	28	67	45–79	3	0.25–14	2	1–3
PD test-retest	9	3	6	68	54–74	7	2.5–14	2	1–2.5
PD longitudinal	21	6	15	67	47–74	2	0.25–14	1	1–2

H&Y: Hoehn and Yahr stage; f: female; m: male.

### MRI acquisition

All participants underwent brain MRI scans on a 3-Tesla system (Discovery MR750; GE Healthcare) prior to PET examination as part of the initial evaluation and to delineate anatomic brain regions of interests (ROI). The T1-weighted sequence has 176 slices of 1 mm thickness, field of view 256 × 256 mm, resolution 1 × 1 × 1 mm, inversion time 450 ms, echo time 3.18 ms, and repetition time 8.16 ms.

### PET acquisition

The radioligand [^18^F]FE-PE2I was prepared with the methods described previously.^15^ Dynamic PET measurements were obtained using a high-resolution research tomograph (HRRT) system (Siemens Medical Solutions). A 6-min transmission scan with a Cs-137 source was performed for attenuation correction. [^18^F]FE-PE2I was injected as i.v. bolus over 10 s, and the catheter was flushed with 10 mL NaCl. Emission data were acquired in list mode over 93 min. PET data were reconstructed in 37 frames of increasing duration (8 × 10 s, 5 × 20 s, 4 × 30 s, 4 × 60 s, 4 × 180 s, 12 × 360 s) using three-dimensional ordinary Poisson ordered subset expectation maximization with 10 iterations and 16 subsets including modelling of the system’s point spread function.^16^ Frame-to-frame motion correction of reconstructed images was applied as previously described.^17^ Two subgroups of PD patients underwent a second [^18^F]FE-PE2I PET examination with the average scan interval of 12 ± 8 days (test–retest cohort) or of 2.3 ± 0.5 years (longitudinal cohort).

### Image analysis and DAT quantification

Image processing and analysis were performed using an in-house pipeline named Solena written in MATLAB (MATLAB r2014b, The MathWorks, Inc.). Within Solena, T1-weighted MP-RAGE sequences of each individual were segmented with FreeSurfer (FreeSurfer v6.0.0, https://surfer.nmr.mgh.harvard.edu/)^18^ and the generated segmentation masks were used to define ROIs of the caudate nucleus, putamen, accumbens area and cerebellum as the reference region. A Functional MRI of the Brain Software Library template (https://fsl.fmrib.ox.ac.uk/fsl/fslwiki/Atlases/striatumconn) was used for the delineation of the sensorimotor striatum (SMS). An in-house developed template was used for the delineation of the substantia nigra (SN).^19^ Subsequently, MRI and dynamic PET images were co-registered.

For DAT quantification, regional dynamic PET data were analyzed with the simplified reference tissue model (SRTM) using the cerebellum as reference region to estimate distribution volume ratio (*DVR*).^20,21^ *SUVR* was computed based on 50–80 minutes and 15–45 minutes of PET data.^8^

### Correction of regional SUVR

To calculate *SUVR*_C_, *k*_2,ref_ = 0.10 min^−1^ (median *k*′_2_ SRTM across brain regions and subjects) ^22^, R_1_ = 1 was used, β_ref_ was estimated using a single exponential fit to 50–80 minutes of cerebellar time activity curve, and β_tar_ was computed using the regression model (equation (2)) with a = 0.0024 min^−1^ in both HC and PD cohort. The volume-weighted average of the caudate and putamen served as the composite region as previously described.^10^

### Statistical analysis

All analyses were performed using the statistics software R (R version 4.3.2, https://www.r-project.org/). We use descriptive statistics such as means with standard deviations or medians with range for continuous variables.

#### Cross-sectional cohort and control subjects

Linear regression analysis and $r^{2}$ were used to assess correlations between parameters. The bias, defined as a measure of the percentage difference between *SUVR* or *SUVR*c, and *DVR* was calculated using the following formula and group differences were evaluated with a paired t-test (*p < *0.017 with Bonferroni correction for multiple comparison).

$$\mathrm{Bias}\;(\%)=\frac{\left|\mathrm{Parameter}\right|-\left|\mathrm{DVR}\right|}{\;\left|\mathrm{DVR}\right|}\times 100$$

Coefficient of variance (CV) of outcome measures were calculated as standard deviation divided by mean of each group. Lin’s concordance coefficient (LCC) was used to evaluate the agreement between two continuous variables by measuring both precision and accuracy. The formula for LCC is:

$$\mathrm{LCC}=\frac{\;2\rho \sigma_{x}\sigma_{y}}{\sigma_{x}^{2}+\sigma_{y}^{2}+(\mu_{x}-\mu_{y}{)}^{2}\;}$$

Where ρ is the Pearson correlation coefficient, $\sigma_{x}$ and $\sigma_{y}$ are the standard deviations of the two variables, and $\mu_{x}$ and $\mu_{y}$ are their respective means. Additionally, Bland-Altman plots were used to visualize the agreement between two methods by plotting the difference between the two measurements against their mean. Cohen’s effect size *d* was estimated to assess the ability of each parameter to differentiate PD patients and HC. The symbol μ represents the mean of each group. Pooled standard deviation (σ) combines the variability of both groups into a single measure.

$$\mathrm{Cohe}n^{\prime }s\;d=\frac{{µ}_{HC}-{µ}_{PD}}{\mathrm{pooled}\;\sigma }$$

Receiver operating characteristic (ROC) analysis was performed to assess the diagnostic performance of the parameters, with the area under the ROC curve (AUC) used as a metric for comparison between methods.

#### Test-retest cohort

For test-retest cohort, test-retest variability (TRV) was calculated as

$$\mathrm{TRV}=\frac{\left|{\mathrm{Parameter}}^{\mathrm{PET}1}-{\mathrm{Parameter}}^{\mathrm{PET}2}\right|}{\frac{1}{2}({\mathrm{Parameter}}^{\mathrm{PET}1}+{\mathrm{Parameter}}^{\mathrm{PET}2})}\times 100$$

#### Longitudinal cohort

Annual longitudinal percent change (ALC) of each parameter was calculated using the formula below, considering the interval between the two PET scans.

$$\mathrm{ALC}=\frac{{\mathrm{Parameter}}^{\mathrm{PET}1}-\;{\mathrm{Parameter}}^{\mathrm{PET}2}}{\begin{array}{c} {\mathrm{Parameter}}^{\mathrm{PET}1}\\ \times \mathrm{interval}\;\mathrm{between}\;\mathrm{baseline}\;\mathrm{and}\;\mathrm{follow}-up\;(y) \end{array}}\;\times 100$$

## Results

### Part I: Validation in cross-sectional cohort

#### Comparison between SUVRc_50–80 min_ and SUVR_50–80 min_

**Figure 1.**
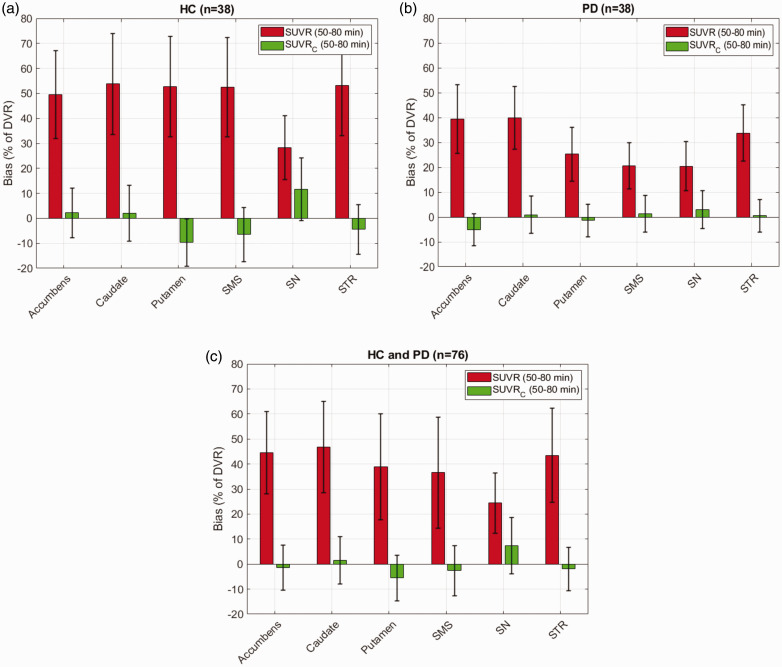
Performance of the correction formula (*SUVR*_C_) in reducing the bias and standard deviation of bias in *DVR* estimation in different regions for [^18^F]FE-PE2I compared to raw *SUVR* values calculated between 50–80 min post injection. Results reported separately for 38 healthy controls (HC, a) and 38 patients diagnosed with Parkinson’s disease (PD, b) and combined (c). SMS: Sensorimotor striatum; SN: Substantia Nigra; STR: Striatum.

**Figure 2.**
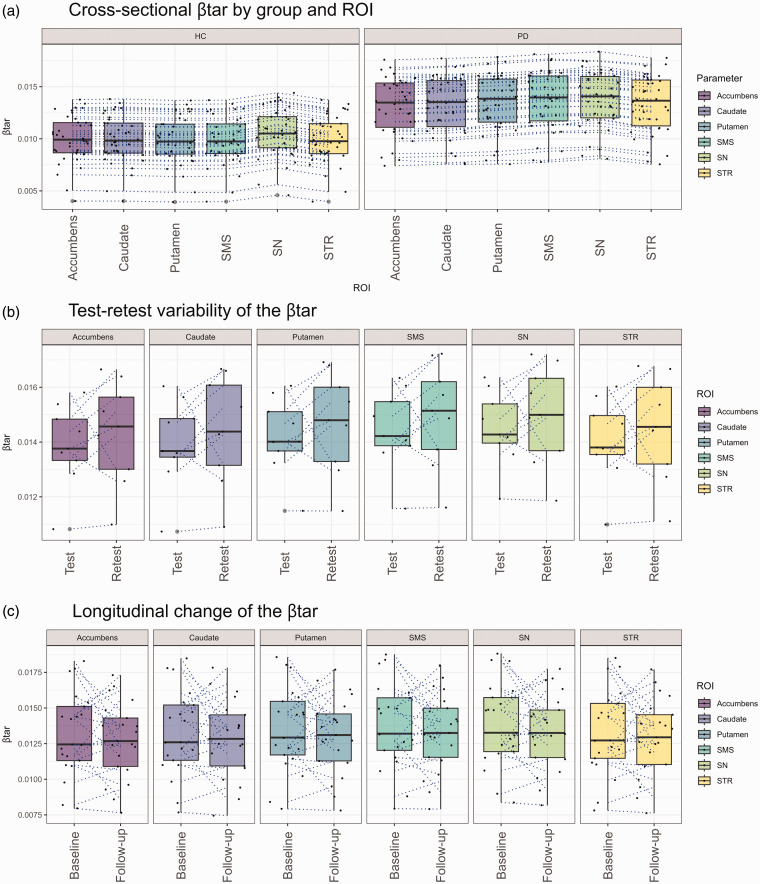
Boxplots illustrate the cross-sectional (a), test-retest (b) and 2-year longitudinal change (c) of the β_tar_ Dotted lines represent individual trajectories between ROIs (a) test and retest (b) or baseline and follow-up (c). SMS: Sensorimotor striatum; SN: Substantia Nigra; STR: Striatum.

Bias and standard deviation of bias in *SUVR*c_50–80 min_ with respect to *DVR* (gold standard) in different regions, compared to *SUVR*_50–80 min_, are shown in Figure 1 and Table 2. *SUVR*_50–80 min_ consistently overestimated the *DVR* across all regions in HC (mean: 48.31% range: 28.32–53.80%) and in the PD group (mean: 29.91% range: 20.45–39.80%). A much lower bias was observed for *SUVR*c_50–80 min_ across all regions in HC (mean: −0.80% range: −9.69–11.64%, *p < *0.001) and in the PD group (mean: −0.12% range: −5.04–2.97%, *p < *0.001). Additionally, the standard deviation of the bias across regions was also smaller for *SUVR*c_50–80 min_ (in HC : 12.72%, in PD : 7.41%) compared to *SUVR*_50–80 min_ (in HC : 20.49%, in PD : 13.95%), except for the SN. As shown in Figure 2, the range of β_tar_ values was between 0.010 and 0.011 min^−1^ (mean ± SD: 0.010 ± 0.002 min^−1^) across all regions in the HC group and between 0.013 and 0.014 min^−1^ (mean ± SD: 0.013 ± 0.003 min^−1^) in the PD group.

**Table 2.** (a) Mean bias (+SD) of *SUVR*_50–80  min_, *SUVR*c_50–80  min_ and *SUVR*_15–45  min_ for *DVR* estimation; (b) Coefficient of variance (%) of outcome measures in HC and PD; (c) Effect sizes of outcome measures in different regions; (d) AUC and confidence interval (CI) determined by ROC analysis.

(a)																		
	HC (n = 38)									PD (n = 38)								
	*SUVR*(50–80 min)		*SUVR*c(50–80 min)		*SUVR*(15–45 min)		p*	p**	p***	*SUVR*(50–80 min)		*SUVR*c(50–80 min)		*SUVR*(15–45 min)		p*	p**	p***
	meanbias	SD	meanbias	SD	meanbias	SD				meanbias	SD	meanbias	SD	meanbias	SD			
Accumbens	49.43	17.56	2.15	9.87	−8.81	8.87	**<0.001**	**<0.001**	**<0.001**	39.49	13.82	−5.04	6.41	−4.37	9.77	**<0.001**	**<0.001**	0.738
Caudate	53.80	20.25	2.03	11.10	−7.90	10.57	**<0.001**	**<0.001**	**0.003**	39.8	12.61	0.94	7.56	−0.41	10.10	**<0.001**	**<0.001**	0.580
Putamen	52.72	20.00	−9.69	9.53	−11.49	9.72	**<0.001**	**<0.001**	0.538	25.25	10.91	−1.42	6.49	6.08	6.06	**<0.001**	**<0.001**	**<0.001**
SMS	52.45	19.81	−6.50	10.92	−10.95	11.36	**<0.001**	**<0.001**	0.156	20.68	9.27	1.31	7.33	5.98	6.60	**<0.001**	**<0.001**	0.034
SN	28.32	12.80	11.64	12.59	9.86	6.97	**<0.001**	**<0.001**	0.537	20.45	9.96	2.97	7.64	7.83	5.88	**<0.001**	**<0.001**	**0.016**
STR	53.11	19.93	−4.44	9.96	−10.06	9.82	**<0.001**	**<0.001**	0.066	33.78	11.34	0.49	6.63	2.58	7.57	**<0.001**	**<0.001**	0.330
Total	48.31	20.49	−0.80	12.72	−6.56	12.11	**<0.001**	**<0.001**	**<0.001**	29.91	13.95	−0.12	7.41	2.95	8.85	**<0.001**	**<0.001**	**<0.001**
(b)																		
	HC (n = 38)				PD (n = 38)													
	*DVR*	*SUVR*_50–80min	*SUVR*c_50–80min	*SUVR*_15–45min	*DVR*	*SUVR*_50–80min	*SUVR*c_50–80min	*SUVR*_15–45min										
Accumbens	12.6%	22.4%	16.5%	12.7%	16.2%	21.5%	14.8%	13.5%										
Caudate	16.6%	28.0%	18.8%	17.7%	21.4%	25.4%	18.7%	21.4%										
Putamen	14.3%	26.9%	17.4%	16.2%	17.1%	23.7%	18.0%	18.1%										
SMS	18.0%	28.0%	19.0%	18.5%	16.6%	21.7%	18.2%	19.2%										
SN	8.2%	15.3%	16.1%	9.4%	7.5%	12.6%	10.9%	9.1%										
STR	14.3%	26.4%	17.5%	16.1%	17.5%	22.7%	17.0%	18.2%										
(c)																		
ROI	*DVR*	*SUVR*_50–80min	*SUVR*c_50–80min	*SUVR*_15–45min														
Accumbens	1.18	1.06	1.32	1.06														
Caudate	1.49	1.34	1.49	1.32														
Putamen	1.86	1.71	1.79	1.77														
SMS	1.84	1.74	1.81	1.78														
SN	1.45	1.25	1.31	1.41														
STR	1.79	1.61	1.70	1.66														
(d)																		
ROI	AUC (lower 95% CI -upper 95% CI)				p for DVR vs. *SUVR*_50–80min	p for DVR vs. *SUVR*c_50–80min	p for DVR vs. *SUVR*_15–45min	p for *SUVR*_50–80min vs. *SUVR*c_50–80min	p for *SUVR*_50–80min vs. *SUVR*_15–45min	p for *SUVR*c_50–80min vs. *SUVR*_15–45min								
	DVR	*SUVR*_50–80min	*SUVR*c_50–80min	*SUVR*_15–45min															
Accumbens	0.843 (0.757–0.930)	0.821 (0.728–0.913)	0.914 (0.853–0.975)	0.821 (0.721–0.921)	0.417	0.013	0.525	0.004	0.988	0.042								
Caudate	0.949 (0.905–0.992)	0.937 (0.887–0.986)	0.966 (0.934–0.998)	0.898 (0.831–0.965)	0.389	0.154	0.020	0.068	0.120	0.019								
Putamen	1 (1–1)	1 (1–1)	1 (1–1)	0.999 (0.997–1)	1.000	1.000	0.480	1.000	0.480	0.480								
SMS	1 (1–1)	1 (1–1)	1 (1–1)	0.999 (0.997–1)	1.000	1.000	0.480	1.000	0.480	0.480								
SN	0.929 (0.874–0.985)	0.902 (0.831–0.973)	0.924 (0.862–0.985)	0.919 (0.854–0.984)	0.303	0.825	0.658	0.061	0.656	0.898								
STR	1 (1–1)	0.997 (0.992–1)	1 (1–1)	0.992 (0.979–1)	0.311	1.000	0.208	0.311	0.277	0.208								

* p values between *SUVR*_50–80min and *SUVR*c_50–80 min, **p values between *SUVR*_50–80min and *SUVR*_15–45 min, ***p values between *SUVR*c_50–80min and *SUVR*_15–45 min.

Bold values are statistically significant after post-hoc correction.

SMS: sensorimotor striatum, SN: substantia nigra; STR: striatum.

CI: confidence interval; SMS: sensorimotor striatum; SN: substantia nigra; STR: striatum.

#### Comparison of SUVRc_50–80 min_ and SUVR_15–45 min_

As shown in Table 2(a), in the HC and PD groups both *SUVR*c_50–80 min_ and *SUVR*_15–45 min_ showed reduced bias in all regions (p < 0.001) compared to *SUVR*_50–80 min_. The means bias and standard deviation of bias for *SUVR*_15–45 min_ and *SUVR*c_50–80 min_ across all regions were −6.56 ± 12.11% and −0.80 ± 12.72% in the HC group (p < 0.001), and 2.95 ± 8.85% and −0.12 ± 7.41% in the PD group (p < 0.001), respectively.. The bias of *SUVR*c_50–80 min_ was smaller than the bias of *SUVR*_15–45 min_ for the accumbens and caudate in the HC group (p < 0.001 and p = 0.003, respectively) and for the putamen and SN in the PD group (p < 0.001 and p = 0.016, respectively). In the HC group, the standard deviation of the regional bias (% of *DVR* units) in *SUVR*c_50–80 min_ ranged from 9.53% for the putamen to 12.59% for the SN and in the case of *SUVR*_15–45 min_ it ranged from 6.97% in the SN to 11.36% in SMS. In the PD group, it ranged from 6.41% in the accumbens to 7.64% in the SN in case of *SUVR*c_50–80 min_, whereas it ranged from 5.88% in the SN to 10.1% in the caudate in case of *SUVR*_15–45 min_.

#### Coefficient of variance (CV) of each parameter

In the HC group (Table 2b), the mean CV ranged from 8.2% to 18.0 for *DVR*, from 16.1% to 19.0% for *SUVR*c_50–80 min,_ from 9.4% to 18.5% for *SUVR*_15–45 min_ For the PD group (Table 2b), the mean CV ranged from 7.5% to 21.4 in *DVR*, from 10.9% to 18.7% in *SUVR*c_50–80 min,_ from 9.1% to 21.4% in *SUVR*_15–45 min_.

#### Diagnostic performance of each parameter

Cohen’s *d* for group differences between the cross-sectional cohorts of PD and HC using *DVR*, *SUVR*_50–80 min_, *SUVR*c_50–80 min_, and *SUVR*_15–45 min_ are presented in Table 2c. The effect sizes ranged from 1.18 in accumbens to 1.86 in putamen for *DVR*, from 1.06 in accumbens to 1.74 in SMS for *SUVR*_50–80 min,_ from 1.31 in SN to 1.81 in SMS for *SUVR*c_50–80 min,_ from 1.06 in accumbens to 1.78 in SMS for *SUVR*_15–45 min_.

The AUCs for discriminating between the PD and HC groups across different regions by each parameter are shown in Table 2d and Supplementary Figure 2. The AUCs ranged from 0.843 (95% confidence interval [CI]: 0.757–0.930) in the accumbens to 1.000 (95% CI: 1.000–1.000) in the putamen and SMS for *DVR*, with similar trends observed for other parameters.

#### Correlation and Lin’s concordance coefficient between DVR, SUVR_50–80 min_, SUVRc_50–80 min_ and SUVR_15–45 min_

**Figure 3.**
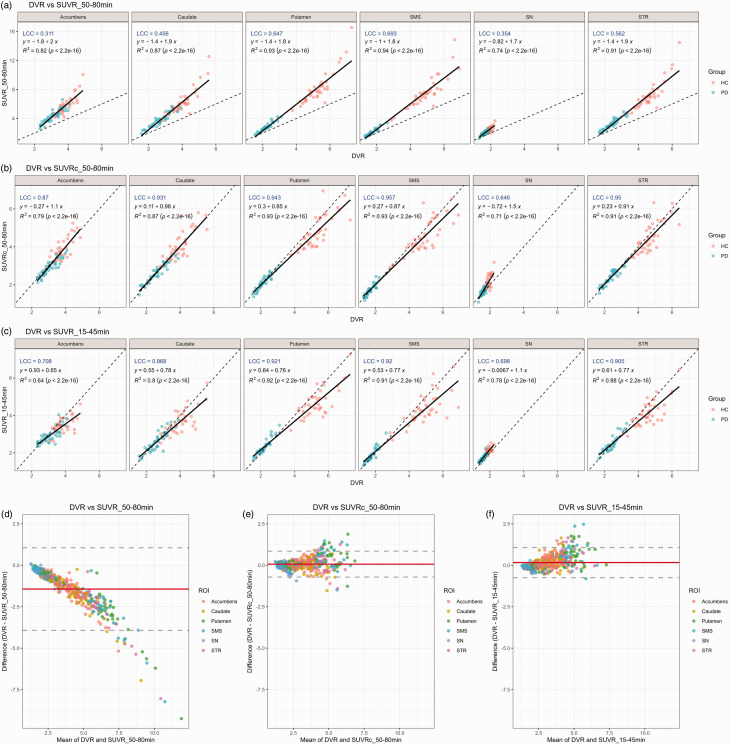
Correlation and Bland-Altman plots between *DVR* and *SUVR*_50–80min (a,d), *SUVR*c_50–80min (b,e) and *SUVR*_15–45min (c,f) by ROI on cross-sectional cohort. SMS: Sensorimotor striatum; SN: Substantia Nigra; STR: Striatum.

The correlation of *SUVR*_50–80 min,_ *SUVR*c_50–80 min_ and *SUVR*_15–45 min_ with *DVR* across all regions is shown in Figure 3a to c. Correlation coefficient (r^2^) across all regions ranged between 0.74 (SN) and 0.93 (putamen) for *SUVR*_50–80 min_, 0.71 (SN) and 0.93 (putamen and SMS) for *SUVRc*_50–80 min_ and between 0.64 (accumbens) and 0.92 (putamen) for *SUVR*_15–45 min_.

The slope ranged from 1.7 in the SN to 2.0 for the accumbens for *SUVR*_50–80 min._ *SUVR*c_50–80 min_ demonstrated a higher slope in the caudate (slope = 0.98) than in the putamen (slope = 0.85), with the highest slope of 1.5 observed in the SN. Conversely, *SUVR*_15–45 min_ showed almost identical slopes in the caudate (slope = 0.78) and in the putamen (slope = 0.76) with the lowest slope of 0.65 observed in the accumbens.

The LCC ranged from 0.311 in the accumbens to 0.693 in SMS for *SUVR*_50–80 min,_ 0.646 in SN to 0.95 in STR for *SUVR*c_50–80 min_, and 0.698 in SN to 0.921 in putamen for *SUVR*_15–45 min._

#### Bland-Altman plots between DVR, SUVR_50–80 min_, SUVRc_50–80 min_ and SUVR_15–45 min_

The Bland-Altman plots in Figure 3d to (f) illustrate the agreement between *DVR* and *SUVR*_50–80 min_, *SUVR*c_50–80 min_, and *SUVR*_15–45  min_ across various brain ROIs, respectively. *SUVR*_50–80 min_ (Figure 3d) showed a downward bias, where the difference (*DVR* - *SUVR*_50–80 min_) decreased as the mean values increase, indicating a systematic overestimation of *SUVR*_50–80 min_ compared to *DVR*, particularly at higher binding levels. Whereas *SUVR*c_50–80 min_ (Figure 3e), and *SUVR*_15–45  min_ (Figure 3f) demonstrated a minimal bias, with most were clustered within the mean difference line (red) and fewer deviations outside the limits of agreement (dashed gray lines). This suggest a better agreement between *DVR* and *SUVR*c_50–80 min_, as well as *SUVR*_15–45  min,_ compared to *SUVR*_50–80 min._

### Part II: Test-retest variability

TRV across all regions are presented in Table 3a. The TRV of the striatum was 3.2 ± 2.5% (*DVR*), 6.4 ± 5.7% (*SUVR*_50–80 min_), 6.8 ± 5.9% (*SUVR*c_50–80 min_) and 3.9 ± 3.2% (*SUVR*_15–45 min_). The mean β_composite_ for test and retest conditions (across subjects) was 0.014 ± 0.002 min^−1^ and 0.015 ± 0.002 min^−1^, respectively (Table 3b). The mean CV of β_composite_ for test and retest conditions were 6.0 ± 3.3% and 9.7 ± 7.1%, respectively. Changes in β_tar_ between test and retest conditions for each ROI are shown in Figure 2b. The mean difference and TRV of β_composite_ were 0.001 ± 0.001 min^−1^ (range: 0.000–0.003) and 9.2 ± 5.1%. The correlations and LCCs are shown in Supplementary Figure 3.

**Table 3.** Test-retest variability (%, n = 9) by region of interest for each outcome measure (a) and for β_composite_ (b).

(a)												
Parameter(n = 9)	Accumbens		Caudate		Putamen		SMS		SN		STR	
	mean	SD	mean	SD	mean	SD	mean	SD	mean	SD	mean	SD
*DVR*	4.1	2.9	4.5	3.4	3.2	2.9	5.1	4.9	4.7	3.6	3.2	2.5
*SUVR*_50–80 min	9.5	6.7	6.5	5.7	8.1	6.4	9.9	8.4	8.3	8.4	6.4	5.7
*SUVR*c_50–80 min	7.1	6.0	8.0	4.8	7.4	6.5	9.0	7.4	8.3	7.7	6.8	5.9
*SUVR*_15–45 min	5.9	4.6	4.1	3.0	3.9	3.7	5.3	4.8	5.8	6.0	3.9	3.2
(b)												
	β_composite_ (min^−1^)				CV_β_composite_ (%)							
	Test	Retest	Difference	TRV (%)	Test	Retest	Difference	TRV (%)				
1	0.011	0.011	0.000	1.1	7.4	8.9	1.5	18.5				
2	0.014	0.015	0.001	5.6	12.3	24.7	12.4	67.1				
3	0.016	0.017	0.001	6.0	4.5	16.3	11.8	113.1				
4	0.014	0.013	0.001	8.1	1.4	6.0	4.6	125.9				
5	0.015	0.016	0.001	8.7	8.6	11.2	2.5	25.7				
6	0.013	0.014	0.001	9.9	5.5	3.0	2.5	59.3				
7	0.015	0.017	0.002	11.4	2.3	4.3	2.1	62.9				
8	0.014	0.015	0.002	12.6	4.8	9.8	4.9	67.2				
9	0.016	0.013	0.003	19.4	6.8	3.1	3.8	75.6				
mean ± SD	0.014 ±0.002	0.015 ±0.002	0.001 ±0.001	9.2 ± 5.1	6.0 ± 3.3	9.7 ± 7.1	5.1 ± 4.1	68.4 ± 35.0				

SMS: sensorimotor striatum; SN: substantia nigra; STR: striatum. Test-retest variability; [abs(Test−Retest)/mean(Test + Retest)] *100 (%).

Difference; [abs(Test-Retest)], TRV: Test-retest variability calculated as [abs(Test-Retest)/mean(Test+Retest)] *100 (%), SD; standard deviation, CV_β_composite;_ % standard error of β_composite_.

### Part III: Longitudinal changes

ALC across all regions is presented in Table 4a. The ALC of the striatum was 4.5 ± 4.2% (*DVR*), 5.2 ± 6.5% (*SUVR*_50–80 min_), 4.4 ± 4.1% (*SUVR*c_50–80 min_) and 4.2 ± 4.1% (*SUVR*_15–45 min_). The mean β_composite_ at baseline and 2-year follow-up was 0.013 ± 0.003 min^−1^ and 0.013 ± 0.003 min^−1^, respectively (Table 4b). Changes in β_tar_ between baseline and 2-year follow-up conditions for each ROI are shown in Figure 2c. The mean difference and ALC of β_composite_ were 0.000 ± 0.002 min^−1^ (range: −0.005–0.005) and −1.9 ± 8.3%. The β_composite_ for the striatum and SN increased in 61.9% (13/21) and 57.1% (12/21) of cases, respectively. The mean ± SD of CV of β_composite_ at baseline and 2-year follow-up were 9.4 ± 4.9% and 8.5 ± 3.4%, respectively. The correlations and LCCs are shown in Supplementary Figure 4.

**Table 4.** Annual longitudinal changes (%, n = 21) by region of interest for each outcome measure (a) and for β_composite_ (b).

(a).												
Parameter	Accumbens		Caudate		Putamen		SMS		SN		STR	
	mean	SD	mean	SD	mean	SD	mean	SD	mean	SD	mean	SD
*DVR*	3.8	4.9	5.3	4.3	4.1	4.2	4.0	4.7	0.4	4.3	4.5	4.2
*SUVR*_50–80 min	4.9	6.9	5.4	6.8	5.0	6.6	4.4	7.2	0.6	6.2	5.2	6.5
*SUVR*c_50–80 min	3.9	4.4	4.4	4.1	4.4	4.5	4.1	5.5	1.1	5.1	4.4	4.1
*SUVR*_15–45 min	2.6	4.4	4.6	5.0	4.0	4.1	3.8	4.8	0.6	4.8	4.2	4.1
(b).												
	β_composite_ (min^−1^)				CV_β_composite_ (%)							
	Baseline	Follow-up	Difference between 2 years	ALC (%)	Baseline	Follow-up	Difference between 2 years	ALC (%)				
mean ± SD	0.013 ± 0.003	0.013 ± 0.003	0.000 ± 0.002	−1.9 ± 8.3	9.4 ± 4.9	8.5 ± 3.4	0.9 ± 6.5	−15.9 ± 55.3				

SMS: sensorimotor striatum; SN: substantia nigra; STR: striatum. Annual Longitudinal percent change, [(PET1-PET2)/PET1]/interval between baseline and follow-up(y)*100 (%).

Difference; (Test-Retest), Annual Longitudinal percent change (ALC); [(Baseline-follow-up)/Baseline]/interval between baseline and follow-up(y)*100 (%), SD; standard deviation, CV_β_composite;_ % standard error of β_composite_.

## Discussion

This study validated a correction method for *SUVR* (*SUVR*c) at late time-window, which accounts for non-equilibrium effects related to tracer clearance, in independent cross-sectional, test-retest and longitudinal (over 2 years) cohorts of HC and PD examined with [^18^F]FE-PE2I PET. In the present study, we extended the time-window to 30 minutes, within the time interval of 50 to 80 minutes.

*SUVR*c values were compared to *DVR* and early *SUVR* (15–45 min), both outcome measures that provide reliable test-retest variability and longitudinal assessment as previously reported^8^. The necessary parameters for the correction formula can be derived from a combination of previous literature estimates and a relatively short range of dynamic PET data during the late time window, enabling straightforward implementation. In the cross-sectional cohort, applying this correction method reduced both bias and variability in corrected *SUVR* quantification in line with previous reports by Honhar et al.^10^

The formula appears to provide robust estimates for different reconstruction methods, ROI templates, and definition of the reference region. *SUVR*_50–80 min_ overestimated *DVR* more in this study than previous report, probably attributed to the later time window that was used. This effect was more pronounced in the HC group than in the PD group, which is expected given that clearance rates are influenced by volume of distribution (*V*_T_) values. β_target_ was higher in the PD group compared with the HC group across all regions (*p > *0.001), which likely contributed to the lower Cohen’s *d* for *SUVR*_50–80 min_, compared to *SUVR*c_50–80 min_.

Compared to *SUVR*_50–80 min_, both *SUVR*c_50–80 min_ and *SUVR*_15–45 min_ reduced the bias in the estimation of *DVR. SUVR*c_50–80 min_ exhibited a stronger correlation compared to *SUVR*_15–45 min_ across all striatal subregions, except in the SN. The linear regression analysis done in relation to *DVR* showed that while the slope of the *SUVR*_15–45 min_ was rather consistent across regions, the slope of the *SUVR*c_50–80 min_ showed more variability across the main striatal subregions, including the caudate and putamen. This variability is likely related to the individual variance of β_tar_ across the ROIs in the cross-sectional cohort, as shown in Figure 2a. Methodological factors, such as inaccuracies in β_tar_ estimation in some regions due to constraints like the linear form of the regression estimator, could explain regional variability in performance of *SUVR*c_50–80 min_. Although it was not statistically significant, the highest β_tar_ was observed in the SN both in HC and PD as expected for a lower DAT binding region.

Test-retest variability of *SUVR*c_50–80 min_ was higher than the test-retest variability of *DVR* and *SUVR*_15–45 min_. Although the numerical difference of β_tar_ between test and retest of the striatum were 0.001 ± 0.001 min^−1^, the test-retest variability was 9.2 ± 5.1% with a range from 1.1 to 19.4%. As equation (1) is more sensitive to errors in β_tar_, even small changes in β_tar_ could lead to increase bias (Supplementary Figure 5).

To mitigate errors in β_tar_, the clearance rate was determined through linear regression based on the radiotracer clearance rate from a composite region instead of estimating it directly from the noisy time-activity curve during a relatively short *SUVR* time window of 20 min. In the present study, we extended the time window to 30 minutes, within the time interval of 50 to 80 minutes. Yet, β_tar_ remains vulnerable to minor changes in the time-activity curve of the composite region. Additional evaluation is needed to ensure the stability of β_tar_ between the test and retest PET examinations.

In longitudinal data, the mean and SD in ALC of *SUVR*c_50–80 min_ was similar to the mean and SD in ALC of *DVR* and *SUVR*_15–45 min_, while *SUVR*_50–80 min_ showed the highest standard deviation. The mean ALC of *SUVR*_50–80 min_ was similar to the mean ALC of *DVR*, *SUVR*c and *SUVR*_15–45 min_, but the SD of ALC of *SUVR*_50–80 min_ was higher than SD of all the other outcome measures. The ALC of β_tar_ was −1.9 ± 8.3% on average which is smaller than ALC of *DVR* or *SUVRc*_50–80 min_ suggesting that changes in β_tar_ have low impact on changes in *SUVR*c.

About two-thirds of PD subjects showed an increase in β_tar_ at follow-up which is expected for reduced DAT binding. There have been no previous studies on the longitudinal changes in [18F]FE-PE2I clearance rates in the striatum and SN. Cross-sectional analysis showed that PD patients had higher β_tar_ than healthy controls, as shown in Figure 2a. However, in about one-third of the longitudinal cohort, β_tar_ decreases, which is likely due to errors in estimating this variable. Additional efforts should be done in order to stabilize β_tar_ for longitudinal assessment of DAT availability using *SUVR*c.

It is important to note that the regression-based estimator (equation (2)) is an empirical construct, and the derivation of the *SUVR* correction formula (equation (1)) is independent of it. Several strategies can be explored in future to further improve the estimate of β_tar_, and thereby improve the accuracy of *SUVR* correction. First, our current regression estimator approximates a simple one-parameter linear relationship between β_tar_ of a region and the inverse of its *SUVR*. For some regions where radiotracer kinetics are markedly different (such as SN for [^18^F]FE-PE2I), this regression estimator may benefit from the inclusion of a quadratic term, with a second constant (slope of quadratic term) that would need to be estimated from pilot data. Alternately, as the reference region and composite region represent two extremes of radiotracer uptake, another strategy could be to obtain an additional estimate for β_tar_ by replacing the composite region by the reference region in the regression estimator and generating a final estimate for β_tar_ by proportionately combining the two estimates based on the *SUVR* of the target region. This could ensure a greater role of the reference region (low binding region) in the estimation of β_tar_ of other low binding regions. Second, Bayesian strategies with a stronger prior for β_tar_ in regions where the current regression estimator leads to higher errors could lead to better estimation without the need to alter the form of the estimator. Finally, strategies involving deep neural networks could be employed to predict regional (or voxel-wise) β_tar_ values from β_composite_ and *SUVR* images. These strategies, either alone or in combination, have the potential to further refine the *SUVR* correction approach.

Validation of the *SUVR* correction can provide useful insights for its translation to other radiotracers. First, this approach is useful for PET tracers used as diagnostic markers, such as amyloid and tau imaging, considering that determining positivity or negativity has a significant clinical impact^23^ and that short scans are approved for clinical purpose.^24,25^ Furthermore, about 10% of the amyloid PET scans showed equivocal findings which is partially explained by suboptimal static imaging time due to individual variance of tissue clearance.^26,27^ The approach presented in this study could also be suitable for PET tracers with slower kinetic properties than [^18^F]FE-PE2I or for PET tracers for which a wide range of time-windows is used across centers (for instance, tau imaging), with the expectation that a slower clearance of the tracer might lead to a more reliable estimation of β_tar_.^28^ Second, this approach could be important for achieving more accurate quantification as a prognostic marker. For example, lecanemab, an FDA-approved disease-modifying drug for early Alzheimer's disease, has demonstrated a reduction in amyloid burden on PET, as measured in centiloids derived from *SUVR*, in a trial substudy. ^29^ Therefore, this *SUVR* correction method could be helpful for ensuring accurate measurement of *SUVR*, which is crucial for assessing longitudinal changes and monitoring treatment efficacy.

## Conclusions

This study validated a correction method for *SUVR* that accounts for non-equilibrium effects related to tracer clearance in a diverse set of cohorts, including cross-sectional, longitudinal, and test-retest groups examined with [^18^F]FE-PE2I PET. The necessary parameters for the correction can be derived from a combination of existing literature estimates and a brief dynamic PET scan during the late time window, facilitating straightforward implementation. In the cross-sectional cohort, employing this correction method minimizes both bias and variability in corrected *SUVR* quantification. Although the correction method was associated with larger test-retest variability than *DVR*, the mean and SD of longitudinal changes in *SUVR*c were similar to those in *DVR*. The method proposed in this study can be used in cross-sectional studies with [^18^F]FE-PE2I, but its use in longitudinal studies should be considered with caution, considering the sensitivity of *SUVR*c to errors in β_tar_. .

## Supplemental Material

sj-pdf-1-jcb-10.1177_0271678X251322407 - Supplemental material for Correcting *SUVR* bias by accounting for radiotracer clearance in tissue: A validation study with [^18^F]FE-PE2I PET in cross-sectional, test-retest and longitudinal cohorts Supplemental material, sj-pdf-1-jcb-10.1177_0271678X251322407 for Correcting *SUVR* bias by accounting for radiotracer clearance in tissue: A validation study with [^18^F]FE-PE2I PET in cross-sectional, test-retest and longitudinal cohorts by Minyoung Oh, Praveen Honhar, Richard E Carson, Ansel T Hillmer and Andrea Varrone in Journal of Cerebral Blood Flow & Metabolism

## Data Availability

Data will be provided upon reasonable request to the corresponding authors.
