# Supplementary material for: Correcting SUVR bias by accounting for radiotracer clearance in tissue: A validation study with [18F]FE-PE2I PET in cross-sectional, test-retest and longitudinal cohorts
Source: J Cereb Blood Flow Metab. 2025 Feb 21;45(7):1357–70. doi: 10.1177/0271678X251322407 (PMC11846093; doi:10.1177/0271678X251322407)

Effect of blood-flow changes on bias in SUVR 15-45 min and SUVRc 50-80 min: Simulations

Unlike DVR, SUVR values are influenced by blood flow, which can conflate their interpretation as markers of target protein density. While this effect is likely more pronounced for early time-windows (such as SUVR 15-45 min), changes in SUVR may reflect blood-flow changes and not just DAT availability changes, and this sensitivity furthers depend on clinical and neurological history, disease status etc. This is an especially important consideration for estimating longitudinal changes in populations with co-morbidities that may influence blood-flow. Recent research with this [18F]FE-PE2I PET has also shown 9-10% lowered blood-flow in PD.[3] To tease-apart the influence of blood flow on these measures, we performed simulations for [18F]FE-PE2I dynamics in the putamen, in both healthy controls and Parkinson's disease participants (typical participants representing our cohort, Hoehn Yahr: 1-2), where we held the DAT level (BP_{ND}) constant, but allowed for gradual blood-flow loss (varying R1 from 1 to 0.75). The results for this simulation, in terms of percent bias in SUVR 15-45 and SUVRc 50-80 is shown in the Figure below. Note that SUVRc computation formula first used R1 = 1 (as it would be impossible to know R1 from PET data during just the 50-80 min time-window), but we also included a case where the correction formula utilized an estimated value of R1 (SUVRc: With R1 estimate). In the latter case, R1 was estimated as average time activity (0-2 min) of target to reference region and could be useful for protocols where an initial 2 min scan is followed by an extended break for the participant before data is recollected for SUVR time-window. Where applicable, the simulations made use of kinetic parameters, and a fixed reference region time activity curve based on a previous publication from our lab[4].

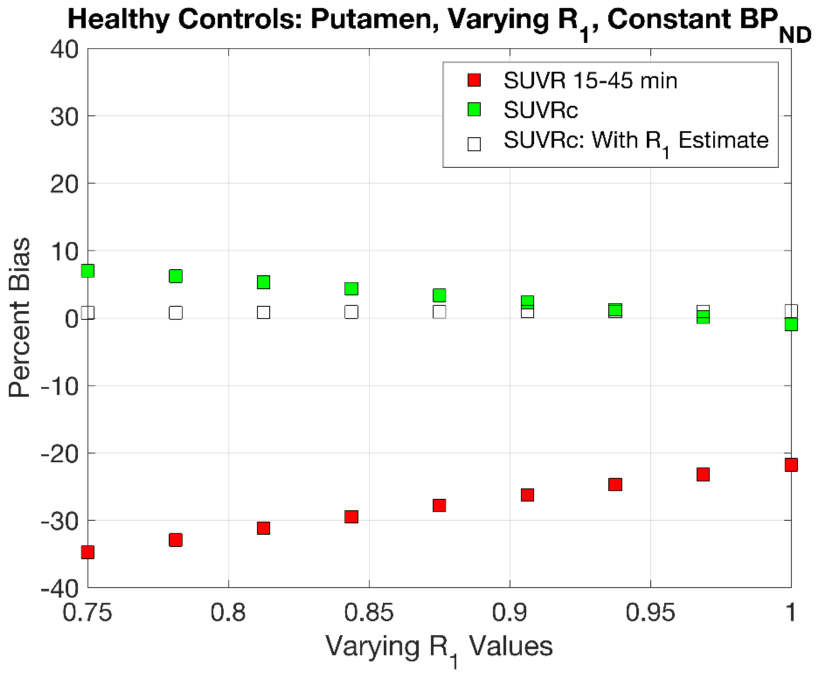

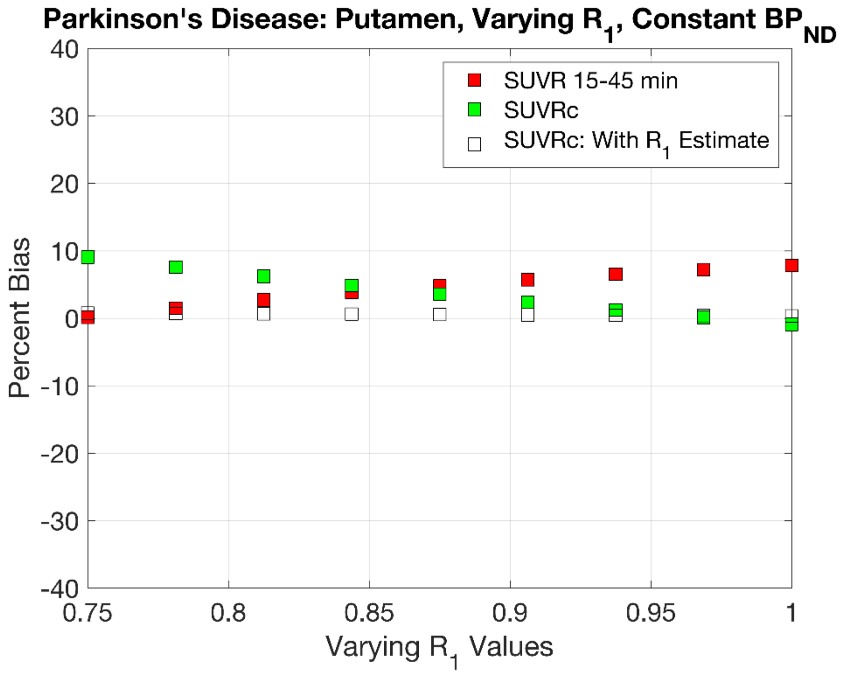

Supplementary Figure 1. Simulations of [^{18}F]FE-PE2I dynamics in the putamen of healthy controls (top) and PD participants (bottom) with varying blood-flow (R1) but constant DAT availability (BP_{ND} = 4.41 for controls, 1.39 for PD based on Fazio et al.[19]) leading to different percent biases in simplified outcome measures (bias defined with respect to *DVR*). For these simulations, we utilized the average reference region time activity curve (Fazio et al.[19]) and forward simulations for putamen time-activity curves was performed using SRTM with varying R1 values. Percent Bias in $SUVRc_{50\text{-}80min}$ was computed using (1) a fixed value of R1 = 1 in the correction formula (solid green), (2) an estimated value of R1 from an additional early 2 min imaging window (hollow squares) and compared to bias in $SUVR_{15\text{-}45min}$ (solid red).

The following observations can be made from this simulation:

(a) Indeed, the bias in SUVR 15-45 depends on the population, with SUVR 15-45 in healthy controls showing a more negative bias, compared to PD.

(b) There is a marked effect of lowered blood-flow on the outcome measures. The effect is more pronounced in SUVR 15-45 for controls, compared to PD population.

(c) While the effect of lowered blood-flow on SUVRc 50-80 is smaller (esp in controls), this can be completely mitigated by collecting initial early time-data to better estimate R1.

Other Supplementary Materials

Supplementary Figure 2. ROC curves for each ROI by parameters.

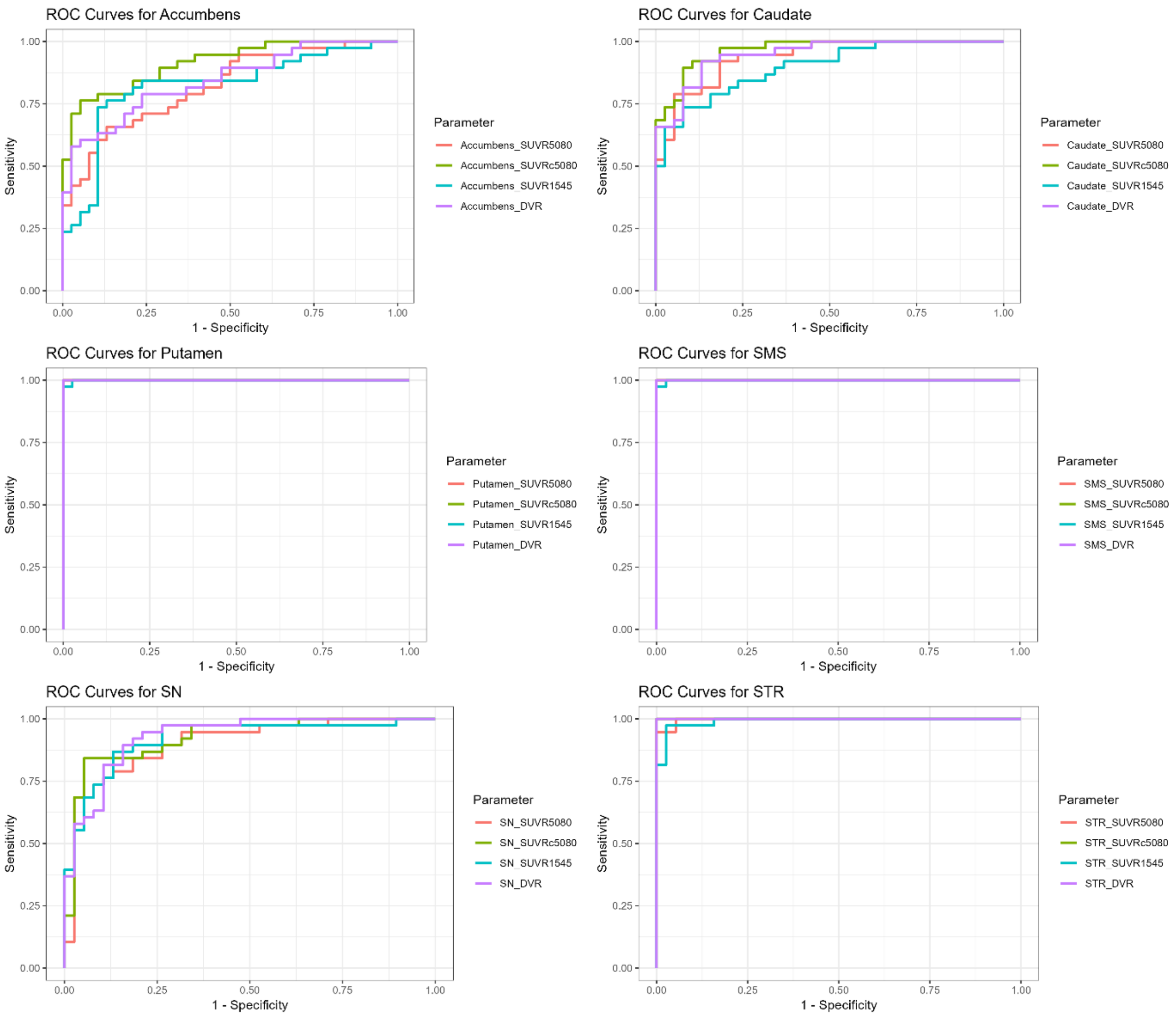

Supplementary Figure 3. The correlations and Lin's concordance coefficient between *DVR* and *SUVR*_50-80min (a), *SUVR*c_50-80min (b) and *SUVR*_15-45min (c) by ROI of test-retest cohort. SMS: Sensorimotor striatum; SN: Substantia Nigra; STR: Striatum

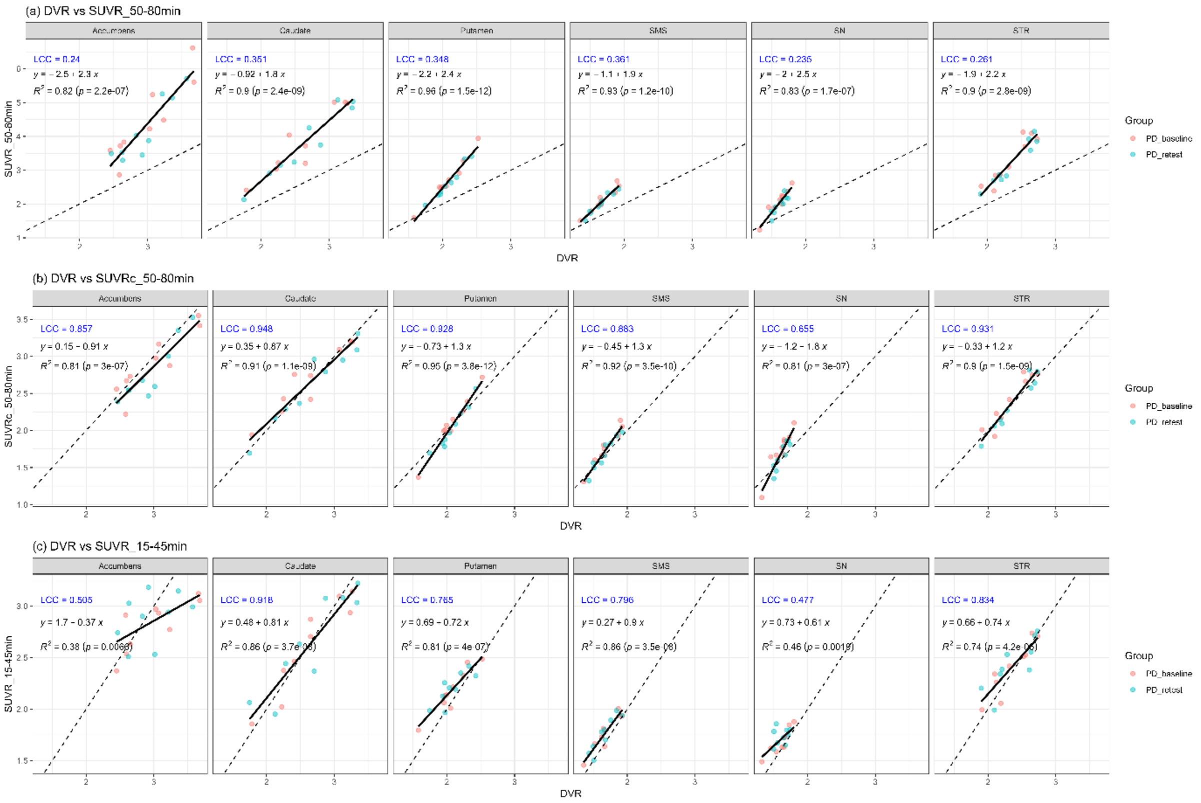

Supplementary Figure 4. The correlations and Lin's concordance coefficients between *DVR* and *SUVR*_50-80min (a), *SUVR*c_50-80min (b) and *SUVR*_15-45min (c) by ROI of longitudinal cohort. SMS: Sensorimotor striatum; SN: Substantia Nigra; STR: Striatum

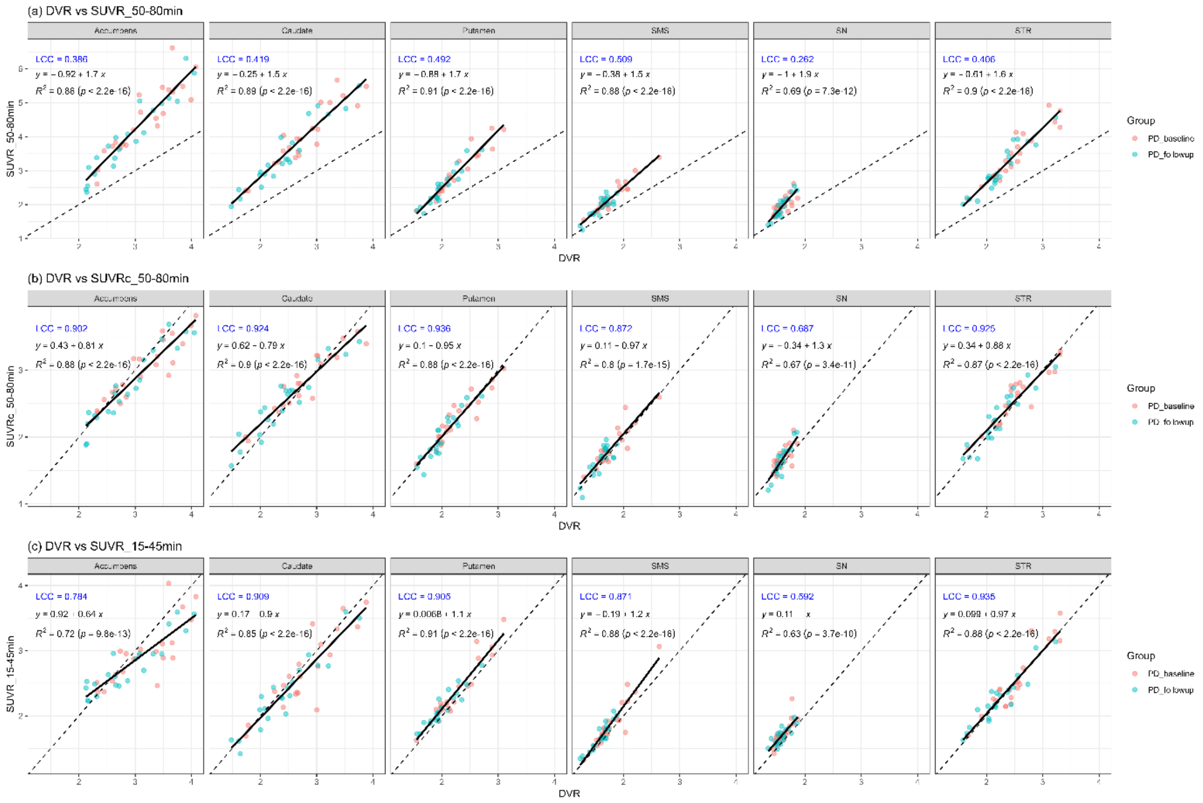

Supplementary Figure 5. Effect of changes in β_{tar} on SUVRc for the cases of HC (DVR: 3.77, SUVR: 5.11, SUVRc: 3.62, β_{tar}: 0.010) and PD (DVR: 1.89, SUVR: 2.23, SUVRc: 2.20, β_{tar}: 0.013)

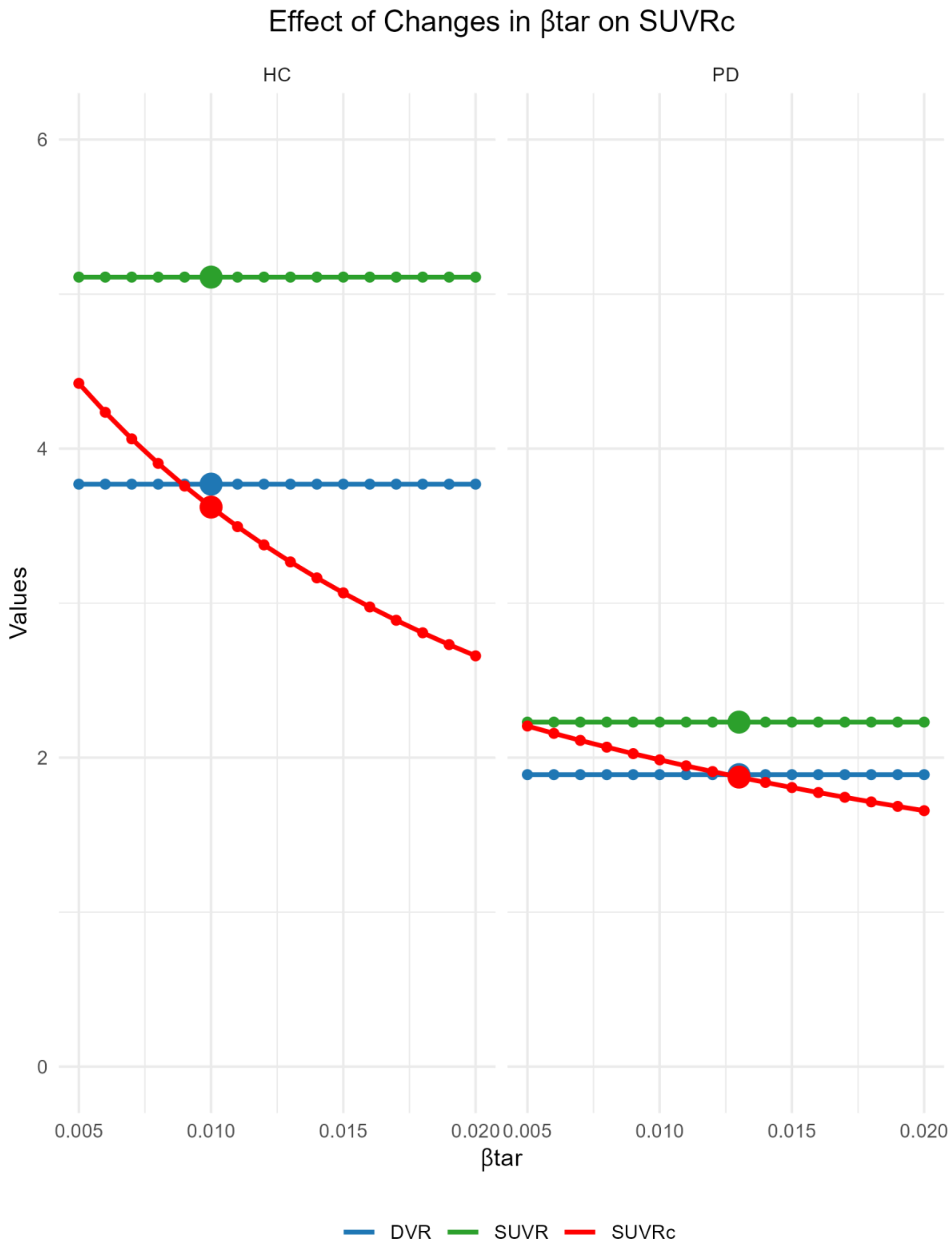

Supplement: sj-pdf-1-jcb-10.1177_0271678X251322407 - Supplemental material for Correcting SUVR bias by accounting for radiotracer clearance in tissue: A validation study with [18F]FE-PE2I PET in cross-sectional, test-retest and longitudinal cohorts [file sj-pdf-1-jcb-10.1177_0271678X251322407.pdf]
